# Sensory Image of Kiwiberry Juice Blended with Apple Juice: Cognitive and Hedonic Insights

**DOI:** 10.3390/foods14223906

**Published:** 2025-11-15

**Authors:** Eliza Kostyra, Anna Piotrowska, Daniel Knysak, Wacław Laskowski, Piotr Latocha

**Affiliations:** 1 Department of Functional and Ecological Food, Warsaw University of Life Sciences—SGGW, 02-787 Warsaw, Poland; 2 Department of Food Market Research and Consumption, Warsaw University of Life Sciences—SGGW, 02-787 Warsaw, Poland; 3 Department of Environmental Protection and Dendrology, Warsaw University of Life Sciences—SGGW, 02-787 Warsaw, Poland; piotr_latocha@sggw.edu.pl

**Keywords:** fruit juices, sensory profile, emotions, FaceReader, consumer liking

## Abstract

Sensory evaluation, based on cognitive and hedonic dimensions, is crucial in developing innovative foods, such as juices made from less popular but highly nutritious fruits. It enables the creation of products with sensory images tailored to consumers’ needs. The research aimed to determine the optimal sensory image of kiwiberry juice blended with ‘Jonagold’ apple juice (10–50%) which would lead to a positive consumer perception. For this purpose, similarities and differences in the sensory profile of kiwiberry juice blended in various proportions with apple juice were evaluated. In hedonic tests, the degree of liking of the products and the analysis of changes in the type and level of consumer emotional reactions (FaceReader) to the juices were performed. To gain a deeper understanding of consumer perceptions, the assessment included expected liking, experienced liking, and purchase intention. It was found that different proportions of apple juice added to kiwiberry juice evoked positive changes in sensory characteristics in the quantitative and qualitative dimensions. The highest sensory quality was noted in the kiwiberry juice with 50% apple juice. Different levels of apple juice addition also conditioned positive changes in affective responses of consumers. Both experienced liking and purchase intention increased with higher levels of apple juice addition (30–50%). The results of emotional reactions reveal that the ranges of variability among examined emotions varied. The most prominent reaction was *neutrality*, followed by *anger*, *happiness*, *disgust*, and *surprise*. An affinity of the degree of liking and emotions to specific juices was noted. The liking attributes were related positively to the sensory image of products with higher content of apple juice and inversely related to the properties of kiwiberry juice which caused negative emotions.

## 1. Introduction

The literature highlights that natural fruit juices are part of the daily diet of consumers across different age groups and provide a deep connection to health due to their nutritional value, functionality, and therapeutic qualities. The consumption of juices is linked to a reduced risk of various diseases, including cancer, cardiovascular diseases, and neurodegenerative disorders [1,2].

The growing trend among consumers towards a healthy diet and lifestyle has prompted the food industry to produce innovative juices. Consumers are now more likely to choose fruit or concentrated juices, as well as traditional and regional products, due to their distinct characteristics resulting from specific production methods, unique composition or place of origin. In recent years, the most significant trends on the juice market have included functional juices and those made from unusual fruits (e.g., noni, pomegranate, sea buckthorn). Additionally, there has been an increase in demand for freshly squeezed, cold-pressed, fermented, and unpasteurized juices [3,4,5,6,7,8,9]. The sensory characteristics of some fruits can limit their use in juice production. One effective solution is to blend these juices with milder-flavoured ones. Combining juices in this way achieves a balanced flavour and sweet-to-sour ratio, creating more palatable products that can help increase consumer demand. Combining different juices can also enhance the final product’s nutritional value and health properties [10,11].

From the perspective of hedonic research, it is important to understand factors affecting consumers’ liking to predict their choice and eating behaviour, thereby ensuring successful product introduction and competitiveness in the marketplace [12]. It is underlying that the expectation plays an important role in food consumption. The expectation may enhance or degrade the perception of the product, even before it is tasted. According to Cardello and Sawyer [13], two types of expectation can be found: a sensory-based expectation or a hedonic-based expectation. In the first type, expectation leads the consumer to believe that the product will possess certain sensory characteristics and influences subsequent perception during consumption. The second type of expectation is based on the hedonic dimension associated with like/dislike to a certain degree of the product by consumers.

A better understanding of product quality and liking is based on integrating sensory perception cues, such as perceived intensities of taste and flavour, with acceptance tests. The relationship between intensity and liking is not evident since it varies with individual sensory descriptors and type of food product being evaluated [14]. It is worth noting that consumer behaviours are associated not only with complex cognitive processing of multisensory perceptions but also emotional experiences with what they eat or drink [15,16].

Emotion plays an important role in consumer decision making. Therefore, it is crucial to understand which product characteristics evoke positive and negative emotions, as well as to determine the relationship between sensory properties, liking, and consumer choices [17,18,19,20]. Research conducted by Jaeger et al. [21] showed that sensory characteristics can be linked to emotions that differ in nature (from positive to negative) and/or degree of arousal (from activation to deactivation), indicating individual differences in how consumers respond emotionally to the sensory attributes of products. Negative emotions experienced by consumers during consumption may result in, among other things, a lack of interest and reluctance to consume the products again, leading to a search for alternative products that will meet their expectations in terms of sensory, hedonic, and emotional aspects. Positive emotions, on the other hand, may motivate consumers to incur higher costs when shopping [22]. The literature emphasises that knowledge and understanding of the mechanisms of emotional reactions can help create products that align with the expectations of potential consumers [23]. Facial expression (FE) is another approach to measure emotions evoked by food/beverage and juices [24,25,26]. It is considered that facial expression analysis can help detect rapid, uncontrolled micro-expressions that have influenced acceptance and preferences [27].

Considering current juice consumption trends, new versions of juices based on kiwiberry fruits have been proposed. Consumer interest in kiwiberry has been steadily increasing. The expanding popularity of this fruit results from its sensory characteristics as well as its high nutritional value. Kiwiberry fruits contain a range of bioactive compounds with antioxidant, anti-tumour, and anti-inflammatory properties [28]. The methodological approach employed in this study, based on cognitive and hedonic dimensions, attempts to explain the sensory characteristics of the examined juices that influence consumer liking and emotional reactions.

The objectives of the study were (1) to determine the similarities and differences in the sensory characteristics of kiwiberry juice blended in various proportions with Jonagold apple juice, (2) to investigate consumer liking of these products, and (3) to analyse the changes in the type and level of emotional reactions of consumers towards the juices. We have evaluated expected liking and experienced liking, as well as purchase intention, to gain a deeper understanding of consumers’ perceptions. Further research directions were also identified.

## 2. Materials and Methods

### 2.1. The Research Material

The kiwiberry juice (not from concentrate, NFC) was made from the cultivar ‘Weiki’ of fruit, one of the most popular *Actinidia arguta* varieties grown in Europe. The fruits were harvested from a commercial orchard (Pyzdry, Wielkopolskie Voivodeship, the western part of Poland in September 2018) at the harvesting maturity stage (6.5 ± 7.5°Brix). Then, the fruits were sorted and stored in a cold chamber at 1 °C with 90% relative humidity for three weeks. They were ripened at 20 °C for several days until they reached eating maturity (soft fruit, ~18°Brix) [29]. The ripe kiwiberry fruits, without damage, were used to produce the juices. After careful selection, the fruits were cold-pressed and pasteurised.

The specific sensory characteristics of kiwiberry juice, such as its relatively high intensity of sour taste and astringent sensation, limit its consumption potential. Therefore, various amounts of selected apple juice were added to kiwiberry juices. The producer of kiwiberry juice provided juices made from six apple cultivars: ‘Rajka’, ‘Champion’, ‘Red Champion’, ‘Golden Delicious’, ‘Gold Bochemia’, and ‘Jonagold’ (supplied by fruit farm Pyzdry, Poland). The apple juices were assessed in preliminary research using the profiling method, which allowed for the selection of apple juice for further testing with kiwiberry juice according to the adopted criteria. The juice obtained from the ‘Jonagold’ apple cultivar had the highest intensity of sweet taste, the lowest level of sour taste, and also exhibited the highest overall sensory quality among the evaluated samples. To obtain the target test samples, kiwiberry juice (KBJ) was blended in the following proportions with ‘Jonagold’ apple juice (JAJ): 90%/10%, 80%/20%, 70%/30%, 60%/40%, 50%/50%, respectively.

### 2.2. Methods

#### 2.2.1. Sensory Analytical Method

The sensory characteristics of the juices were performed using Quantitative Descriptive Analysis (profiling method). Following the method procedure, samples were prepared in the preliminary stage for the selection of attributes by the expert panel, which were then discussed [30,31]. In the next stage of the procedure, the distinguishing features were compiled, and their definitions were established. Two training sessions were performed (total durations 5 h).

The assessment of the juice samples was carried out according to twenty-two defined descriptors, including odour attributes (kiwiberry, apple, green, sour, sweet nectar, refreshing, sharp), texture cues (density, viscidity, fruit pulp particles) and taste/flavour traits (kiwiberry, apple, green, sour, sweet, astringency, bitter, persistent, refreshing, sour aftertaste, sweet aftertaste), as well as overall sensory quality. The intensity of the descriptors in the juices was measured on a linear scale (0–10 cm) anchored at extremes “none” (on the left of the scale) to “very strong” (on the right of the scale). Overall sensory quality was defined as the impression of the harmony of all attributes, with no or only slight intensity of negative notes.

The sensory profiling of juices was performed by a panel of 10 assessors qualified as experts according to ISO 8586:2014 [32]. The panel had 18 years of methodological experience (theoretical and practical) in profiling assessments of different products. Each sample was evaluated in two independent sessions; the average results were based on 20 individual results.

#### 2.2.2. Consumer Tests

Consumers between 18 and 21 years of age performed the juices assessment (*F* = 90%, *M* = 10%). They were recruited on the university campus based on their availability and interest in participating in the study. Participants declared a consumption of fruit juices and stated buying and drinking practices, e.g., apple and multi-fruit juices, naturally cloudy, pressed juices, and one-day juices.

First, kiwiberry juice and a blend of kiwiberry juice with ‘Jonagold’ apple juice were evaluated by 50 participants in a semi-consumer study using *FaceReader* (FR). All participants were informed about the camera recording before the study began. Those who agreed to the requirements participated in the test. Subsequently, consumer research on the juices was conducted by 100 people using a scaling method. Participants who took part in the FR measurement also performed hedonic tests on the juices. The interval between the *FaceReader* study and the consumer rating was two weeks. All participants agreed that the sensory evaluation data could be used for research purposes. A written informed consent form was prepared and signed by each participant before their involvement in the study.

*FaceReader research*. Emotions from the juices were measured using *FaceReader 6* (Noldus Information Technology, Wageningen, Netherlands). The software allowed for the assessment and analysis of consumer reactions by recording the intensity and type of emotions over time: *neutral*, *happy*, *sad*, *angry*, *surprised*, *scared*, and *disgusted*. The software automatically measured the intensity of emotions on a scale from 0 (no emotion) to 1 (maximum intensity of emotion) [33].

Participants were informed about the measurement procedure using *FaceReader* before starting the sample evaluation. The sessions were coordinated by individuals with experience in FR analysis. During the study, the camera was positioned slightly below the participant’s eye level, under the guidelines provided in the instructions. The distance between the respondent and the camera was 50–60 cm. To obtain the best image quality, the participants’ faces were additionally illuminated with two USB LED lamps (placed on both sides of the monitor) to eliminate reflections/shadows. A ‘general’ face model was selected for the study. The recordings from the study were saved and analysed frame by frame using FR software (version 6).

The tests were conducted individually with each participant on pre-arranged days and at pre-arranged times. Before starting the analysis, each person was asked to take a seat. After hearing the start signal, the participant drank the full contents of the first given sample. Emotional reactions were measured for 30 s from the moment the participant placed the juice sample in the mouth. A stop signal informed them that the analysis was complete. The testing time for the sample was determined in preliminary research; the measurement of emotions concerning the set of samples took approximately 8–10 min for each participant.

*Scaling method*. Consumer research was performed to determine the expected level of liking for the juices (before tasting, evaluation based on the associations related to odour, taste, flavour, and consistency), the level of liking for the odour, taste/flavour, consistency, and the experienced liking (after tasting). The research was conducted using a nine-point hedonic scale [34]. Odour, taste/flavour, consistency, and the experienced liking were marked on the scale: dislike extremely = 1, neither like/nor dislike = 5, like extremely = 9. Additionally, consumers were asked to indicate their purchase intention for each sample (scale: 1 = would definitely not buy, 5 = neither buy/nor not buy, 9 = would definitely buy).

### 2.3. Sample Preparation and Presentation, Testing Conditions

The juices were prepared the day before the evaluations according to a standard procedure developed during preliminary tests [29]. All juice samples were stored in a refrigerator (6–8 °C) until sensory and consumer tests were carried out. In the profiling assessment and consumer tests, the sets of six samples containing 30 mL of juice (of the appropriate variant) were prepared for each assessor. In the *FaceReader* study, participants received 10 mL of each juice sample (for one sip).

The juice samples were served monadically for evaluation at room temperature (21 ± 2 °C). The juices were poured into plastic containers (100 mL) and covered with a lid. In the *FaceReader* study, juice samples were presented in 50 mL containers. The taste and flavour of the samples were neutralised with still water. The sample sets for each panellist were individually coded and presented for sensory evaluation in random order to avoid the carry-over effect.

All assessments of the juices were performed in a sensory laboratory fulfilling the general requirements of the ISO 8589:2007 standard for testing conditions [35]. The laboratory was equipped with 10 individual booths and a computerised software for data acquisition (ANALSENS, version 7.5). The evaluations were carried out during the morning and early afternoon hours. The consumer tests were conducted following the pre-established rating schedule.

### 2.4. Statistical Analysis

Two-way ANOVA with interaction was performed to determine the significance of differences in attribute intensity between the juices evaluated, considering products, assessors, and interactions as fixed variables (sensory profiling). Means were compared using Fisher’s LSD significant test. Principal Component Analysis (PCA) was used to determine similarities and differences in the sensory characteristics of juices. PCA was performed on the covariance matrix (Cov-PCA). To examine the differences between juices in terms of liking attributes and purchase intention, a two-way analysis of variance (ANOVA) was used. The significance of differences between samples for the features was determined using Fisher’s LSD significant difference test. The obtained results were statistically analysed with XLSTATS version 2021 software by Addinsoft (Paris, France). For all tests, *p* < 0.05 was considered significant.

To explore the impact of juice types (variants) on emotional variability in consumers tested with *FaceReader* and to check the variability of liking and purchase intention, we analysed the results using Kohonen’s neural networks. The effectiveness of individual classifications was assessed by performing a variance analysis. Classifications with five clusters were included to explain the variability of the attributes of liking and the intention to purchase juices by consumers. They were characterised by explaining more than half of the total measure of variability (the calculated correlation coefficient for each attribute assessed about liking ranged from 0.6 to 0.9, with an average of 0.8). Classifications with seven clusters were adopted as they met the expectations in terms of the degree of explanation of consumer emotion variability. They were characterised by explaining more than half of the total variability measure (the calculated correlation coefficient for each emotion type ranged from 0.2 to 0.9, with an average of 0.7). Cross-tabulation and chi-square tests were performed to check whether the type of juice tested impacted the cluster to which it belonged. The analyses were performed using the software Statistica (StatSoft, Inc., 2014, version 13.1, Krakow, Poland).

Pearson correlation coefficients and Multiple Factor Analysis (MFA) were used to determine similarities and differences in the sensory characteristics of juices, their relationship with consumer liking assessment, and emotional consumer response. The Pearson correlation and MFA were performed using the R program version 4.2.2 [36], with the use of RStudio version 0.99.896 [37].

## 3. Results

### 3.1. Sensory Properties of the Kiwiberry Juice and Blends of Kiwiberry Juice with ‘Jonagold’ Apple Juice

The results of the descriptive analysis of the kiwiberry juice and blends of kiwiberry juice with ‘Jonagold’ apple juice are presented in Table 1. Statistically significant differences in the intensity of the attributes between samples were found for three odour attributes, three consistency descriptors, eight taste/flavour cues, two aftertaste feelings, and overall sensory quality. It is essential to note that the juice with 90% kiwiberry content revealed very similar sensory characteristics to the 100% kiwiberry juice (except for the intensity of the sour odour and sour taste). The higher percentage of apple juice (20–50%) in the examined samples caused a significant increase in the intensity of the apple odour compared to 100% kiwiberry juice. It was found that the intensity of sour odour considerably decreased with apple juice addition, while the perceptibility of the sweet-nectar attribute increased. There was no significant effect of different proportions of kiwiberry juice and apple juice on the intensity of kiwiberry, green, refreshing, and sharp odour. In texture characteristics, 100% kiwiberry juice was determined to be viscous and quite dense, with the perceptibility of pulp particles. The results indicated that samples with higher levels of apple juice (30–50%) were significantly less dense and viscous and also represented a lower sensation of fruit pulp particles compared to 100% KBJ. The intensity of sour taste and kiwiberry flavour was most pronounced at 100% kiwiberry juice. The higher amount of apple juice in the kiwiberry sample significantly decreased the level of kiwiberry flavour (40–50% JAJ), sour taste (10–50% JAJ), green flavour (20–50% JAJ), bitter taste (40–50% JAJ), astringency (30–50% JAJ), and persistent impression (40–50% JAJ) as opposed to sweet taste and apple flavour. There were no statistical differences in the refreshing sensation among the examined juices. It was found that the feeling of sour aftertaste dominated over sweet aftertaste in samples. The higher level of apple juice addition to kiwiberry ones (30–50% JAJ) caused a significantly lower intensity of sour aftertaste and a higher sensation of sweet aftertaste.

The differences in the intensity of odour, consistency, flavour, and taste attributes in juices influenced the overall sensory quality of the examined juices, which increased significantly with the percentage of apple juice in the kiwiberry juice. Adding at least 20% apple juice positively influenced the sensory characteristics of the juices.

**Figure 1:**
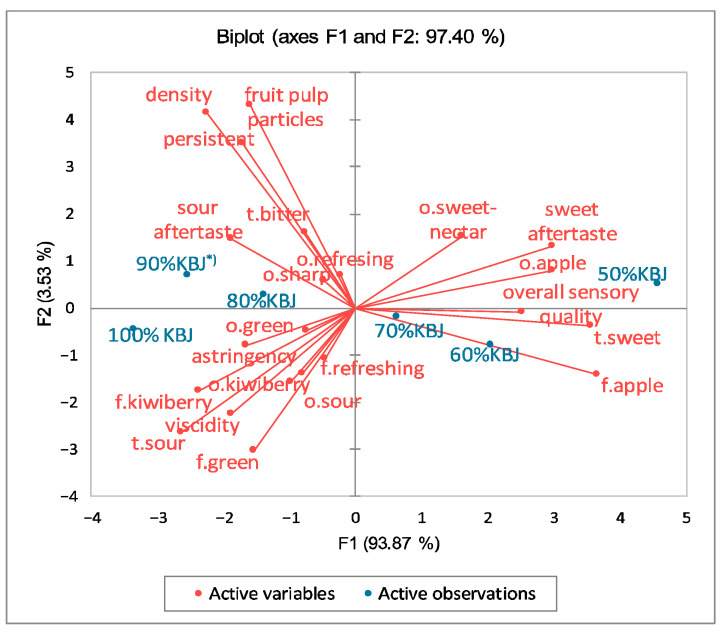
Principal component analysis plot of the similarities and differences in the sensory characteristics of the kiwiberry juice (KBJ) and blends of kiwiberry/’Jonagold’ apple juice (JAJ); *^)^ Samples: 90%KBJ/10%JAJ, 80%KBJ/20%JAJ, 70%KBJ/30%JAJ, 60%KBJ/40%JAJ, 50%KBJ/50%JAJ; o.—odour, t.—taste, f.—flavour.

The results of the descriptive analysis of the examined juices are presented in Figure 1 as a PCA biplot. A total 93.87% of the variability was related to the first principal component, whereas the second principal component accounted for 3.53% of the variability. Samples of the juices are distributed along the first principal component, from left to right, of the PCA. The samples with 30%, 40%, and 50% apple juice addition are located near the sensory overall quality and the attributes such as apple flavour and odour, sweet-nectar odour, sweet taste, and sweet aftertaste. On the opposite side of the PCA, samples with 80–100% kiwiberry juice content are situated closely to astringency impression, sour aftertaste, green odour, sharp odour, refreshing odour, and flavour.

### 3.2. Changes in the Level of Liking for Kiwiberry Juice and Blends of Kiwiberry Juice with ‘Jonagold’ Apple Juice

The results of consumer evaluation concerning expected liking, odour, taste/flavour, and texture liking, as well as experienced liking and purchase intention for kiwiberry juice and its blend with ‘Jonagold’ apple juice, are presented in Table 2. The juices did not differ significantly in expected liking. Notable changes between the samples were observed in odour, taste/flavour, consistency, experienced liking, and purchase intention. The 100% kiwiberry juice received a substantially lower rating for odour liking compared to the sample containing 50% apple juice addition. Significant differences in taste/flavour liking were noted between 100%KBJ and other samples evaluated. In subsequent juices, the level of liking increased significantly; however, there were no differences in taste/flavour liking scores among examined variants with 70%, 60%, and 50% content of kiwiberry juice. Regarding consistency, the increasing addition of apple juice in blends with KBJ also resulted in a higher hedonic score. Significant changes in consistency liking occurred between 100% kiwiberry juice and the blends with 20–50% apple juice addition.

Analysis of the results indicated that the experienced liking significantly increased with the addition of apple juice to the KBJ sample. However, 30–50% level of apple juice in the blends with kiwiberry sample did not cause significant changes in the level of experienced liking. Very similar results were obtained for consumers’ intention to buy.

**Figure 2:**
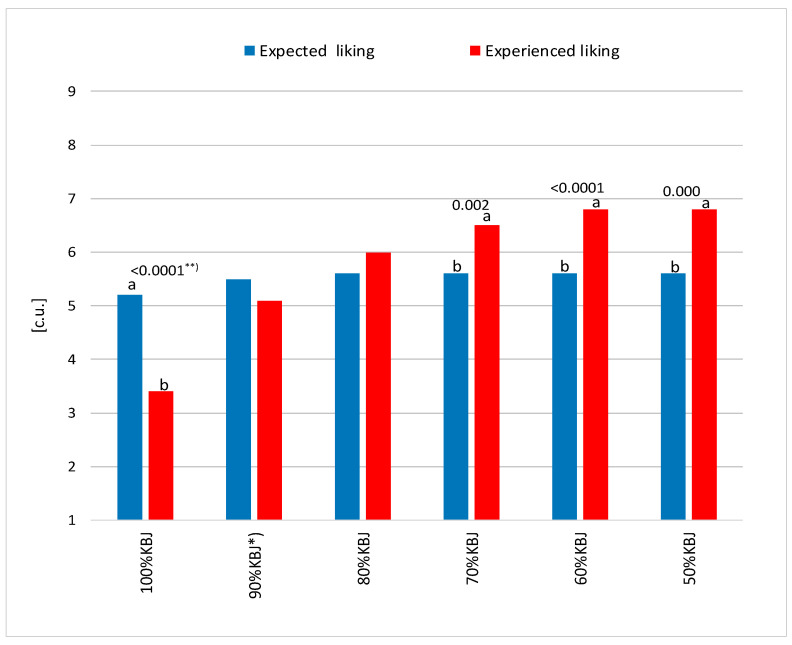
Relationship between the expected and experienced liking for kiwiberry juice (KBJ) and blends of kiwiberry with ‘Jonagold’ apple juice (JAJ). The average values marked with different letters in the graph differ significantly for a given juice variant at *p* ≤ 0.05; **^)^ *p*-Value; *^)^ Samples: 90%KBJ/10%JAJ, 80%KBJ/20%JAJ, 70%KBJ/30%JAJ, 60%KBJ/40%JAJ, 50%KBJ/50%JAJ.

The differences between the expected and experienced liking of the juices are shown in Figure 2. A clear distance in the mean results can be observed between the expected and experienced liking in the 100%KBJ sample. In this case, expected liking remained statistically higher than experienced liking. It is noteworthy that a 10% and 20% addition of Jonagold apple juice to KBJ positively nullified the statistical differences between expected and experienced liking. On the other hand, a subsequent higher proportion of apple juice in the samples (30–50%) caused a significant increase in the level of experienced liking, affecting the distance (difference) in the evaluation compared to the expected liking. In these juices, the experienced liking was significantly higher than expected liking.

**Figure 3:**
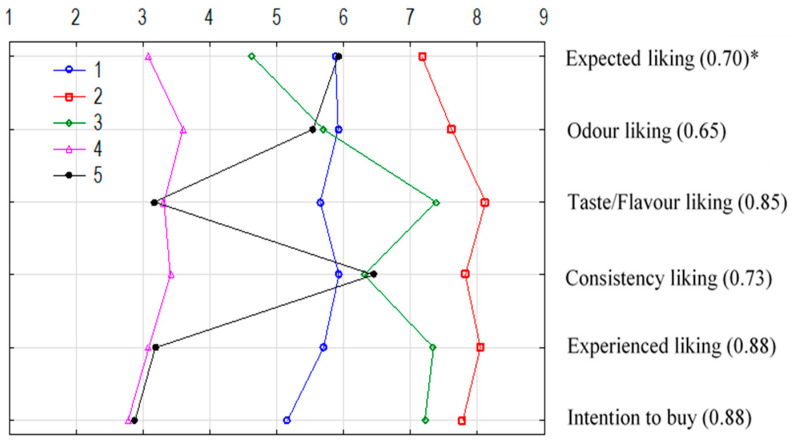
Profiles of the five clusters of liking and intention to buy of examined juices; * correlation ratio.

An exploratory classification was performed to extract clusters for explanation and description of profiles of variation in consumer evaluations of juices (Figure 3). The proportion of profiles in the examined juices is shown in Table 3.

Five cluster profiles were distinguished in the experiment. The characteristic feature of cluster 1 was a moderate level of liking for the rated attributes in juices and a medium score for purchase intention of the samples. The affinity of cluster 1 with 80% (47.3%) and 90% (44.6%) kiwiberry juice content was noted. Cluster 2 was characterised by high ratings for the evaluated attributes (expected liking, odour, taste/flavour, consistency, experienced liking, and purchase intention) and was more strongly associated with 50%KBJ/50%JAJ (40.9%). The characteristics of cluster 3 were linked to higher values for taste/flavour liking, experienced liking, and purchase intention compared to other attributes. Cluster 3 exhibited an affinity for 30–40% apple juice content (22.7%). The results indicated that cluster 4 had low scores for all attributes, while cluster 5 displayed low scores for taste/flavour liking, experienced liking, and intention to buy. The latter two clusters (4 and 5) had a higher affinity for 100% kiwiberry juice (26.4% and 46.4%, respectively). The calculated correlation ratio, shown in Figure 3, is different and relatively high, indicating that the classification of clusters was effective regarding variability for each analysed characteristic (attribute).

### 3.3. Determining the Type and Level of Emotions Evoked by the Flavour of Kiwiberry Juice and Blends of Kiwiberry Juice with ‘Jonagold’ Apple Juice Using FaceReader

The results of emotional reactions measured with *FaceReader* related to the evaluated juices are shown in Table 4. The type and level of emotion showed that the ranges of variation in each type of emotion varied. Among all facial expressions, *neutrality* was the most prominent in the examined juices. However, we can observe that *anger* and *happiness* represented higher intensity than the other emotions, in particular *scared* and *sad*, which were at the margins of the overall emotional reactions in the samples.

The mimic state of neutral occurred from the lowest amount in 100%KBJ to the highest score in the sample with 80%KBJ. The highest level of *happiness* was recorded in 70%KBJ/30%JAJ. With an increasing amount of apple juice added to the samples, no regular changes in the level of *happiness* were observed. For example, 100%KBJ and the sample with 90% kiwiberry juice content represented a higher level of *happiness* than the sample with 60% kiwiberry juice addition. The highest intensity of *sadness* was found in 100% kiwiberry juice and 90%KBJ and the lowest level was in 50% kiwiberry juice. The results indicate that the highest rating of the emotion *disgust* was observed in 100%KBJ and the lowest in the 60% kiwiberry juice content. Similar patterns were observed for the emotion *scared*. In contrast, *angry* emotion increased the most in 60% kiwiberry juice addition, followed by 100% KBJ. When analysing changes in the intensity of the emotion *surprised* between juice variants, it was noted to be highest in 30% and 50% addition of apple juice to the kiwiberry sample. In contrast, the lowest intensity of *surprised* emotion was found in 80%KBJ/20%JAJ (Table 4).

**Figure 4:**
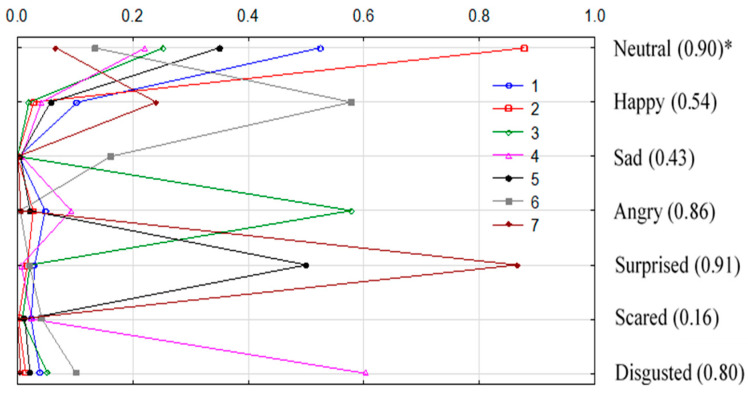
Profiles of seven clusters of consumers’ emotional states; * correlation ratio.

It was stated that seven profiles adequately reflected the emotional reactions of consumers (Figure 4, Table 5).

The reported correlation ratio for the analysed emotions obtained different values and was highest for the facial states of *neutral* and *surprised*, followed by the emotions of *disgusted* and *angry*, indicating that the classification of clusters was effective in terms of variability for the considered characteristics. Cluster 1 was the most significant and covered 48% of the recorded emotional states, while cluster 7 included the smallest amount of reactions (2%). Cluster 1, in addition to the dominant facial state of *neutrality*, was characterised by a slightly higher level of positive emotion (*happiness*) than the other emotional states. The affinity of this cluster was noted for juices containing the addition of apple juice to the kiwiberry sample, particularly in 80%KBJ/20%JAJ (Table 2).

Cluster 2, comprising 20% of the recorded emotional states, contained the highest level of *neutrality* among all clusters. In contrast, cluster 3 (13% of recorded reactions) was characterised by a relatively high intensity of negative emotions like *angry* and showed affinity for 60%KBJ and 100%KBJ. It was found that cluster 4 represented a high intensity of *disgusted* emotion. Cluster 5 was mainly determined by the level of *surprise*. It is noteworthy that cluster 6 was mainly determined by a high intensity of positive emotion (*happiness*) and followed by the state of *sadness*, showing a higher affinity for 70%KBJ/30%JAJ and 100%KBJ. A characteristic feature of cluster 7 was the high proportion of *surprised* and, to a lesser extent, *happiness*, and it showed a higher affinity to 70% kiwiberry sample. It seems that a high level of *happiness* and a relatively low intensity of negative emotions, i.e., *sad*, *angry*, or *disgust*, accompanied the sample with 30% apple juice addition, which caused a relatively higher amount of *happy* emotion represented by the profile of clusters 6 and 7 (Table 5, Figure 4).

### 3.4. Relationships Between Sensory Profile Characteristics and Consumer Research

**Figure 5:**
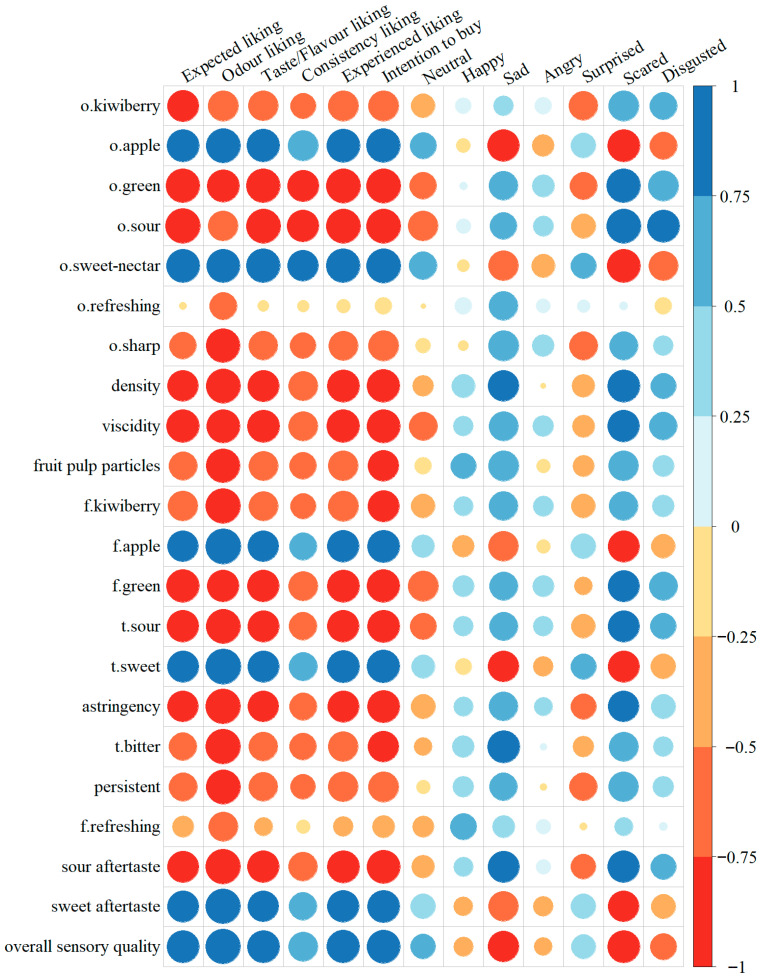
Graphical representation of the Pearson correlation matrix for the sensory characteristics of juices, their consumer liking assessment, and emotional consumer response. Blue and red colours indicate positive and negative correlations, respectively. The lighter the tone used, the less strong is the corresponding correlation; o.—odour, t.—taste, f.—flavour.

Pearson correlation analysis was conducted to explore the relationships between sensory attributes and consumer evaluations, including liking and emotional responses. The aim was to identify the sensory features of fruit juices that influence consumer acceptance. The results are visualised in a heatmap (Figure 5). The analysis revealed that expected liking was strongly positively correlated with apple odour and flavour, sweet-nectar odour, sweet taste and aftertaste, as well as overall sensory quality. In contrast, significant negative correlations were observed for sour odour, taste, and aftertaste; green odour and flavour; density; viscosity; and astringency. Several sensory attributes significantly influenced odour liking. Strong positive correlations were found with apple odour and flavour, sweet-nectar odour, sweet taste and aftertaste, and overall sensory quality. Conversely, strong negative correlations were observed with sharp odour, green odour and flavour, consistency-related attributes (including density, viscosity, and fruit pulp particles), kiwiberry flavour, sour taste and aftertaste, bitter taste, astringency, and persistent sensation. Taste/flavour liking was positively associated with apple odour and flavour, sweet-nectar odour, sweet taste and aftertaste, and overall sensory quality. Strong negative correlations were noted with green odour and flavour; sour odour, taste, and aftertaste; viscosity; density; and astringency. Consistency liking showed strong negative correlations with sour and green odours, while a positive correlation was observed with sweet-nectar odour. Both experienced liking and purchase intention were strongly positively correlated with apple odour and flavour, sweet-nectar odour, sweet taste and aftertaste, and overall sensory quality. Negative correlations were found with green odour and flavour; sour odour, taste, and aftertaste; density; viscosity; and astringency.

In terms of emotional responses, the emotion *scared* was strongly positively correlated with green odour and flavour; sour odour, taste, and aftertaste; as well as viscosity and density. It was negatively correlated with apple odour and flavour, sweet-nectar odour, sweet taste and aftertaste, and overall sensory quality. It was found that the emotion *sad* was negatively associated with apple odour, sweet taste, and overall sensory quality, while positively correlated with density, bitter taste, and sour aftertaste. The emotion *disgusted* showed a strong positive correlation with sour odour. No strong correlations were observed between other emotions (*happy*, *neutral*, *angry*, *surprised*) and the sensory attributes of the juices.

The correlation analysis presented in this study offers valuable insights into the sensory drivers of consumer acceptance and emotional engagement with fruit juices.

**Figure 6:**
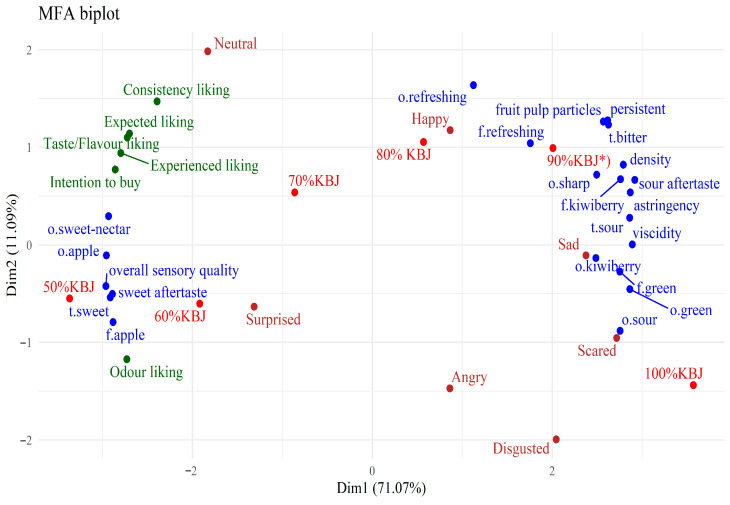
The MFA biplot illustrates similarities and differences in the sensory characteristics of juices (in blue)**,** their relationship with consumer liking assessment (in green), and emotional consumer response (in brown). The position of the six examined juice samples (in red) shows their relation to the above features; Samples: *^)^ 90%KBJ/10%JAJ, 80%KBJ/20%JAJ, 70%KBJ/30%JAJ, 60%KBJ/40%JAJ, 50%KBJ/50%JAJ; o.—odour, t.—taste, f.—flavour.

The results of the MFA on the sensory profiles of the examined juices in relation to the average consumer preferences and emotional reactions are presented in Figure 6. Like in the case of the PCA plot in Figure 1, most of the variability, 71.07%, was related to the first plot dimension, and the second dimension accounted for 11.09% of the variability. The second dimension only weakly separates the attributes positioned on the left side of the first dimension, and thus it is driven mostly by the differences in the attributes on the right side of the plot. According to the plot, all liking scores increase with an increase in the sensory overall quality, sweet-related odours and tastes, as well as the apple odour and flavour. Consequently, these scores are negatively associated with attributes located on the opposite end of the first dimension, bitter, sour, green, sharp, refreshing odours and flavours, astringency, viscidity, density and persistent sensations, and others. It was found that samples with 30% apple juice content were located close to liking attributes. In terms of the second dimension, most of the liking scores are closer to the refreshing odour and flavour, fruit pulp particles, persistent and density sensations, and bitter taste, opposed to the kiwiberry odour, and sour and green odour and flavour. An exception is the consumers’ odour liking, which is positioned on the opposite side of the second dimension compared to the other liking scores.

Figure 6 suggests that the *neutral* and *surprised* emotions are the ones related to the sensory overall quality, and apple and sweet-related odours and tastes. Products with a higher addition of apple juice (40–50%) to kiwiberry samples were relatively close to these attributes. The remaining emotions are shifted in the direction of the bitter, sour, green, sharp, refreshing odours and flavours, astringency, viscidity, density, persistent sensations, and others. The *sad*, *scared*, and *disgusted* emotions are most related with the above attributes. Regarding the second dimension, *neutral* and *happy* emotions seem most associated with the refreshing odour and flavour, fruit pulp particles, persistent and density sensations, and bitter taste. Samples with 10–20% addition of apple juice to kiwiberry samples were found close to these characteristics. The *angry*, *scared,* and *disgusted* emotions are closer to the kiwiberry odour, sour and green odour and flavour, and samples with 100% kiwiberry juice.

## 4. Discussion

The detailed sensory characteristics of juices in the quantitative–qualitative dimension allowed for the determination of the cognitive image of various versions of juices based on kiwiberry combined with apple juice from the ‘Jonagold’ apple variety. In this study, kiwiberry juice was primarily characterised by kiwiberry odour and flavour, accompanied by a sour taste. Green odour and flavour, along with a persistent sensation, were also significant in the juice, and the profile was positively rounded off with a subtle intensity of odour and apple flavour. Our results indicate that the properties of kiwiberry juice were similar to the sensory attributes characterising *Actinidia arguta* (hardy kiwifruit). A study by Wang et al. [38] showed that green odour and flavour, as well as grassy odour and flavour, and fruity odour and flavour (such as melon and watermelon) were present in fresh *Actinidia arguta* fruits, whose peel was characterised by a perceptible bitter and sour taste. Latocha et al. [39], analysing different genotypes of *Actinidia arguta* and their hybrids, found that the fruit represented in the sensory profile a soft, sour, and moderately astringent skin with an additional irritant attribute. It was noted that the taste of the soft, jelly-like pulp of hardy kiwifruit was dominated by fruity flavour and sweet taste, with a low impression of irritating, vinous, astringent, and sour taste. According to Latocha and Jankowski [40] *Actinidia arguta* should represent sweet taste and odour as these attributes are important for most consumers.

In this study, the overall sensory evaluation (the impression of harmony of all attributes) was mostly negatively correlated with the odour and taste attributes characterising kiwiberry juice. The overall sensory evaluation changed in a positive direction under the influence of the addition of different proportions of ‘Jonagold’ apple juice to kiwiberry juice, and it is in line with the study performed by Kostyra et al. [29]. The sensory profile of the juices based on kiwiberry with the addition of apple juice (produced from a blend of different apple cultivars) has become increasingly harmonised (balanced) with higher intensity of apple odour and flavour, sweet taste, and with lower perceptibility of green odour and flavour, sour taste, and astringency, which was also reflected in higher overall liking.

According to sensory research performed by Rødbotten et al. [41], many consumers like fruit juices because of their sweet taste. In apple juice evaluations conducted by Stolzenbach et al. [12], it was found that consumers liked samples with a balanced sweet–sour taste the most. The consumers appreciated such a version of the product, regardless of the intensity of other descriptors, compared to juices perceived as intensely sour and very sweet. In addition, sweet and sour taste turned out to be key attributes in the sensory profile of juices and played an essential role in liking acquisition. In the current study, we observed a similar relationship, where among the blends of kiwiberry juice with increasing amounts of ‘Jonagold’ apple juice, harmonious samples were obtained in the intensity of sweet and sour taste as well as higher liking and purchase intention. The results indicated that sour-tasting juice variants received lower overall liking scores than sweeter juices, which is in agreement with the findings of Sabbe et al. [42] and Rocha and Bolini [43].

The literature emphasises the important role of congruence or qualitative matching (consonance) in the perception of sensory impressions and the creation of an integrated effect, known as flavour or, more broadly, the comprehensive sensory quality of a product. The importance of matching applies to both the cognitive aspect of sensations (qualitative and quantitative dimensions) and the affective (hedonic) evaluation is demonstrated [30]. Analysing our results, it can be concluded that the changing proportions of kiwiberry juice to apple juice determined an increasing consonance, which was reflected in the results of the overall sensory quality (profiling method) and the experienced level of liking (scaling method).

The affective dimension of impressions also refers to emotions related to the sensory properties of the product, playing a role in explaining and complementing the results of hedonic evaluations [44]. Considering that sweet taste generates positive emotions (*happiness*, *surprise*), while bitter taste evokes negative emotions (*anger*, *disgust*) and sour taste determines different emotional associations, such as *surprise* and *sadness*, a study was conducted using *FaceReader*. In the available literature, few studies have been performed with *FaceReader* to measure facial expressions with juices [20,25,26]. The experiment showed that kiwiberry juices with changing percentages of ‘Jonagold’ apple juice elicited different emotional reactions among participants. This finding is also confirmed by the research of Zhi et al. [20] on different juices. It was found that negative emotions *sadness*, *anger*, and *disgust* represented a noticeably high negative correlation tendency to hedonic scores. Similarly, in our study, the kiwiberry juice with a higher level of negative emotions compared to other juice variants had the lowest liking for taste/flavour and experienced liking.

It is important to note that *surprise* can be considered in both a positive and a negative aspect. Depending on the type of juice, positive surprise is likely associated with a perceived harmonisation of sensory impressions (as in the case with the samples containing 30–50% apple juice). In contrast, negative surprise arises from increased consumer arousal, which may be influenced by psychophysical properties (intensity and quality of the stimulus) and/or, as presented in the literature, collative properties relating to attention, perceived complexity, and inconsistency [12]. A negative *surprise* may occur in the case of kiwiberry juice and the variant with the lowest addition of ‘Jonagold’ apple juice, which exhibited a low impression of harmony due to an intense perceptibility of green odour and flavour, sour taste, and astringency sensation. On the other hand, it cannot be discounted that some individuals liked these juice versions due to the emergence of a happy emotion.

In the current study, juice samples elicited an emotional response, probably linked to consumers’ hedonic expectations, as confirmed by research conducted by Danner et al. [25,26]. It should be emphasised that the identified emotional profiles, which account for a significant portion of the variability in consumers’ facial expressions, alongside the cluster profiles alluding to different degrees of liking for the juices, proved to be very valuable. The study found that the sample with 30% of ‘Jonagold’ apple juice triggered a higher level of *happy* emotion than the other variants examined. Research conducted by Desmet [45] demonstrated that consumers can experience various emotions associated with a product, and that food often evokes multiple emotions simultaneously, which is also corroborated by the present study. Consumer emotional cluster profiles revealed a predominance of certain emotions alongside others at a lower level, indicating an affinity for specific juice variants.

Danner et al. [26], in a study using *FaceReader*, divided participants into two groups—one in which consumers showed clearly visible facial reactions when consuming juices (about 75% of participants) and another consisting of people with poker faces showing a low level of emotional reactions (about 25%). In the present study, juices determined a relatively high level of facial neutrality (defined as neither positive nor negative emotions) among consumers. According to Danner et al. [26], the lack of positive emotional reactions may be caused by the weak impact of positive stimuli or be the result of consumers’ concentration and analytical thinking, which may lead to the suppression of positive emotions. When interpreting the results of this study related to *neutrality*, the possibility of participation in the study by people with poker faces should be taken into account. In general, positive emotions, i.e., *happy* and *satisfaction*, are significantly positively correlated with the degree of liking a product, while emotions such as *disgust* are negatively related to the level of liking [46]. These results align with our observations, indicating that attributes detracting from the product’s sensory characteristics were negatively correlated with liking descriptors and triggered adverse emotional reactions.

To determine the optimal product characteristics to maximise the level of liking, it is suggested to study the arousal potential of a product [12,47,48]. The stimulus with arousal above or below the optimal level will be less liked the further it deviates from the optimal designated liking range for the product. This can be illustrated as an inverted U-shaped relationship between degree of liking and arousal level [12]. In the present study, the addition of apple juice in the ratio of 30–50% to kiwiberry juice evoked a significantly higher level of taste/flavour liking and experienced liking. Thus, these variants fell within the optimal liking range for the evaluated samples, especially when compared to the kiwiberry juice and the variant with the lowest addition of ‘Jonagold’ apple juice, which represented the stimuli with arousal above the optimal liking range.

The literature focuses on the relationship between the expected and experienced liking of various products. Expectations play a crucial role in evaluating food products [49]. This study shows that the expected liking was greater than the experienced liking for kiwiberry juice. Probably the discrepancy in consumer expectations resulted in negative disconfirmation (product worse than expected) and the emergence of the contrast effect [13,49]. Therefore, a large difference between the expected and experienced liking for taste and flavour perception can generate unpleasant emotional feelings (e.g., *disgust*). We also observed that a greater proportion of ‘Jonagold’ apple juice in variants did not lead to a further increase in the difference between expected and experienced liking, indicating that an assimilation effect might have occurred (in which consumers adjust their perception of the product to what they expected). According to the “curiosity hypothesis” [50,51], it cannot be ruled out that the juices were perceived as pleasant due to the slight differences in the degree of liking between the expectations.

Research has shown that the sensory properties of products influence consumer satisfaction and consumption and purchasing behaviour [42,52,53,54]. It can be concluded that the relationship between the expected and experienced liking and the propensity to purchase juices depended on the relative proportions of kiwiberry juice and apple juice and determined consumers’ emotional and hedonic responses as a result of their sensory characteristics.

According to the literature, the degree of liking for products also depends on non-sensory factors, including psychological, cultural, and socio-economic influences [55,56] that were not examined in the present study, which can be considered as a limitation of our study.

## 5. Conclusions

The findings indicate that different proportions of ‘Jonagold’ apple juice added to kiwiberry juice led to positive changes in sensory characteristics. The sensory profile of the kiwiberry juices with apple juice remained in consonance (was more balanced) with higher intensity of apple odour and flavour, sweet taste, and with lower intensity of attributes associated with kiwiberry juice, which was also reflected in higher overall liking and purchase intention. An analysis of the relationship between the expected liking and the experienced liking for the examined juices revealed the possibility of both positive disconfirmation (the product turned out to be better than expected) and negative disconfirmation (the product was perceived as worse than expected) along with a contrast effect depending on the proportions of kiwiberry juice and apple juice. The identified emotional profiles helped explain a significant part of the variability in consumers’ facial expressions. They were characterised by the predominance of a particular emotion, with other emotions present at a lower level, along with an affinity for specific juice varieties. Conversely, the cluster profiles of attribute liking and purchase intention provided deeper insights into consumer perceptions. The analysis demonstrates that positive changes in the sensory image of the examined juices in cognitive and hedonic dimensions increased with the addition of apple juice in the blend (30–50%). This study broadens the understanding of consumer perceptions of blended juices aimed at achieving balanced sensory properties. The approach offers valuable insights for producers seeking to develop new versions of blended juices that may enhance the product’s nutritional value and health benefits. The outcome is establishing the optimal sensory image of the juices, which determines positive consumer perception. The optimal proportion of apple juice in kiwiberry juice formulations is 30%. This level of addition ensures a balanced sensory profile, increases consumer liking, and reduces negative emotional responses while maintaining the high nutritional value characteristics of kiwiberry juice.

Future studies could investigate how the addition of alternative fruit juices affects the sensory and emotional characteristics of kiwiberry-based blends. Additionally, expanding the analysis of emotional responses through physiological measures, such as biometric indicators, may provide deeper insights into consumer behaviour. Using focus groups to explore participants’ motivations, attitudes, and opinions about different juice varieties would be highly beneficial and add significant value to the research. Furthermore, research on shelf-life stability and the long-term health effects of regular consumption could aid in developing functional juice formulations that have improved sensory quality and nutritional value.

## Figures and Tables

**Table 1:** Sensory profiling of the kiwiberry juice and kiwiberry blends with ‘Jonagold’ apple juice (*n* = 20).

Attributes	100% KBJ *^)^	90%KBJ/ 10%JAJ	80%KBJ/ 20%JAJ	70%KBJ/ 30%JAJ	60%KBJ/ 40%JAJ	50%KBJ/ 50%JAJ	*p*-Value
o.kiwiberry	4.74	4.24	4.59	4.34	4.15	3.92	0.145
o.apple	2.67 ^d^	3.06 ^cd^	3.55 ^c^	4.37 ^b^	4.34 ^b^	5.24 ^a^	<0.0001
o.green	2.57	2.27	2.30	2.01	2.03	1.89	0.066
o.sour	3.50 ^a^	2.86 ^b^	2.94 ^ab^	2.76 ^b^	2.64 ^b^	2.46 ^b^	0.027
o.sweet-nectar	1.71 ^c^	2.22 ^bc^	2.31 ^bc^	2.75 ^ab^	2.71 ^ab^	3.23 ^a^	0.000
o.refresing	2.96	3.33	3.00	3.03	3.07	2.89	0.486
o.sharp	1.33	1.41	1.34	1.02	1.15	1.01	0.141
density	4.67 ^a^	4.80 ^a^	4.28 ^ab^	3.78 ^bc^	3.00 ^d^	3.17 ^cd^	<0.0001
viscidity	6.08 ^a^	5.60 ^ab^	5.39 ^ab^	5.28 ^b^	4.99 ^bc^	4.28 ^c^	0.000
fruit pulp particles	4.11 ^ab^	4.48 ^a^	4.02 ^ab^	3.68 ^bc^	2.98 ^d^	3.21 ^cd^	<0.0001
f.kiwiberry	5.17 ^a^	5.06 ^a^	4.83 ^ab^	4.48 ^ab^	4.21 ^b^	3.10 ^c^	<0.0001
f.apple	2.65 ^c^	2.73 ^c^	3.16 ^bc^	3.86 ^b^	4.72 ^a^	5.52 ^a^	<0.0001
f.green	3.35 ^a^	2.82 ^ab^	2.57 ^b^	2.73 ^b^	2.43 ^b^	1.73 ^c^	<0.0001
t.sour	5.73 ^a^	5.18 ^b^	5.05 ^b^	4.73 ^bc^	4.32 ^c^	3.29 ^d^	<0.0001
t.sweet	1.31 ^d^	1.44 ^cd^	1.92 ^c^	2.94 ^b^	3.13 ^b^	4.18 ^a^	<0.0001
astringency	3.28 ^a^	3.07 ^ab^	3.01 ^abc^	2.68 ^bc^	2.43 ^cd^	1.85 ^d^	<0.0001
t.bitter	0.86 ^ab^	1.10 ^a^	0.81 ^abc^	0.63 ^bcd^	0.46 ^cd^	0.41 ^d^	0.003
persistent	2.70 ^ab^	2.89 ^a^	2.85 ^a^	2.13 ^bc^	1.62 ^c^	1.65 ^c^	<0.0001
f.refreshing	3.72	3.80	3.66	3.88	3.68	3.27	0.478
sour aftertaste	4.37 ^a^	4.36 ^a^	4.06 ^ab^	3.55 ^bc^	3.24 ^c^	2.96 ^c^	<0.0001
sweet aftertaste	0.46 ^d^	0.74 ^d^	1.04 ^cd^	1.62 ^bc^	1.94 ^b^	3.03 ^a^	<0.0001
overall sensory quality	4.88 ^e^	5.11 ^de^	5.48 ^cd^	6.00 ^bc^	6.35 ^b^	6.98 ^a^	<0.0001

o.—odour; t.—taste; f.—flavour; *^)^ KBJ—kiwiberry juice; JAJ—‘Jonagold’ apple juice; mean values with different letters in rows are significantly different at *p* ≤ 0.05.

**Table 2:** Features liking and intention to buy for kiwiberry juice and blends of kiwiberry juice with ‘Jonagold’ apple juice (*n* = 100).

Cues	100%KBJ *^)^	90%KBJ/ 10%JAJ	80%KBJ/ 20%JAJ	70%KBJ/ 30%JAJ	60%KBJ/ 40%JAJ	50%KBJ/ 50%JAJ	*p*-Value
Expected liking	5.2	5.5	5.6	5.6	5.7	5.7	0.109
Odour liking	5.7 ^b^	5.6 ^b^	5.7 ^b^	5.9 ^ab^	6.0 ^ab^	6.2 ^a^	0.030
Taste/Flavour liking	3.3 ^d^	5.2 ^c^	6.1 ^b^	6.7 ^a^	6.9 ^a^	6.8 ^a^	<0.0001
Consistency liking	5.5 ^c^	5.9 ^bc^	6.3 ^a^	6.3 ^a^	6.3 ^a^	6.2 ^ab^	0.000
Experienced liking	3.5 ^d^	5.1 ^c^	6.0 ^b^	6.5 ^a^	6.8 ^a^	6.8 ^a^	<0.0001
Intention to buy	3.3 ^d^	4.6 ^c^	5.7 ^b^	6.1 ^ab^	6.4 ^a^	6.6 ^a^	<0.0001

*^)^ KBJ—kiwiberry juice; JAJ—Jonagold apple juice; ^a,ab,b,bc,c,d^ mean values with different letters in rows are significantly different at *p* ≤ 0.05.

**Table 3:** The share of profiles of the distinguished clusters in the overall liking of examined juices (Cramer’s V = 0.31) (*n* = 600 total results).

Profiles of the Distinguished Clusters	100%KBJ *^)^	90%KBJ/ 10%JAJ	80%KBJ/ 20%JAJ	70%KBJ/ 30%JAJ	60%KBJ/ 40%JAJ	50%KBJ/ 50%JAJ	Total
1	20.9	44.6	47.3	31.8	25.5	19.1	31.5
2	1.8	10.9	18.2	31.8	37.3	40.9	23.5
3	4.6	9.1	18.2	22.7	22.7	20.9	16.4
4	26.4	18.2	12.7	11.8	11.8	15.5	16.1
5	46.4	17.3	3.6	1.8	2.7	3.6	12.6
Total (%)	100.0	100.0	100.0	100.0	100.0	100.0	100.0

*^)^ KBJ—kiwiberry juice; JAJ —‘Jonagold’ apple juice.

**Table 4:** Intensity of facial expressions elicited by kiwiberry juice and kiwiberry/apple juice blends (*FaceReader*, *n* = 50).

Samples		Emotions						
		Neutral	Happy	Sad	Angry	Surprised	Scared	Disgusted
Total	Mean	0.502	0.090	0.008	0.111	0.075	0.017	0.079
	Median	0.481	0.006	0.000	0.007	0.003	0.000	0.006
100%KBJ *^)^	Mean	0.457	0.091	0.016	0.122	0.069	0.028	0.095
	Median	0.446	0.004	0.000	0.006	0.001	0.000	0.010
90%KBJ/10%JAJ	Mean	0.501	0.098	0.021	0.110	0.080	0.020	0.077
	Median	0.479	0.007	0.000	0.005	0.004	0.000	0.006
80%KBJ/20%JAJ	Mean	0.532	0.085	0.003	0.107	0.049	0.017	0.074
	Median	0.510	0.005	0.000	0.008	0.002	0.000	0.008
70%KBJ/30%JAJ	Mean	0.499	0.113	0.003	0.096	0.088	0.015	0.075
	Median	0.471	0.007	0.000	0.005	0.004	0.000	0.005
60%KBJ/40%JAJ	Mean	0.501	0.072	0.005	0.132	0.080	0.012	0.073
	Median	0.487	0.006	0.000	0.010	0.003	0.000	0.006
50%KBJ/50%JAJ	Mean	0.519	0.084	0.002	0.098	0.085	0.014	0.077
	Median	0.490	0.007	0.000	0.006	0.004	0.000	0.005
*p*-value		<0,01	<0.01	<0.01	<0.01	<0.01	<0.01	<0.01
Correlation ratio		0.09	0.07	0.11	0.06	0.07	0.08	0.04

*^)^ KBJ—kiwiberry Juice; JAJ—‘Jonagold’ apple juice.

**Table 5:** The contribution of clustering profiles in the rating of emotions evoked by the consumption of juices (Cramer’s V = 0.06) (*n* = 138,056 total emotional states observations).

Clusters	100%KBJ *^)^	90%KBJ/ 10%JAJ	80%KBJ/ 20%JAJ	70%KBJ/ 30%JAJ	60%KBJ/ 40%JAJ	50%KBJ/ 50%JAJ	Total
1	46.3	48.2	54.0	44.2	48.9	44.8	47.7
2	15.7	19.8	19.2	21.7	17.5	24.4	19.7
3	14.9	13.5	12.4	10.8	15.0	9.8	12.7
4	8.9	5.5	7.2	7.5	6.7	8.7	7.4
5	7.0	6.0	5.2	7.2	7.6	7.5	6.7
6	5.1	4.5	1.5	5.1	2.2	2.4	3.5
7	2.1	2.6	0.5	3.4	2.2	2.5	2.2
Total (%)	100.0	100.0	100.0	100.0	100.0	100.0	100.0

*^)^ KBJ—kiwiberry juice; JAJ—‘Jonagold’ apple juice.

## Data Availability

The original contributions presented in this study are included in the article. Further inquiries can be directed to the corresponding authors.
